# Effective Zero-Shot Multi-Speaker Text-to-Speech Technique Using Information Perturbation and a Speaker Encoder

**DOI:** 10.3390/s23239591

**Published:** 2023-12-03

**Authors:** Chae-Woon Bang, Chanjun Chun

**Affiliations:** 1 Department of Computer Engineering, Chosun University, Gwangju 61452, Republic of Korea; bcw4045@chosun.ac.kr; 2 Glosori Inc., Gwangju 61472, Republic of Korea

**Keywords:** zero-shot multi-speaker speech synthesis, diffusion model, information perturbation

## Abstract

Speech synthesis is a technology that converts text into speech waveforms. With the development of deep learning, neural network-based speech synthesis technology is being researched in various fields, and the quality of synthesized speech has significantly improved. In particular, Grad-TTS, a speech synthesis model based on the denoising diffusion probabilistic model (DDPM), exhibits high performance in various domains, generates high-quality speech, and supports multi-speaker speech synthesis. However, speech synthesis for an unseen speaker is not possible. Therefore, this study proposes an effective zero-shot multi-speaker speech synthesis model that improves the Grad-TTS structure. The proposed method enables the reception of speaker information from speech references using a pre-trained speaker recognition model. In addition, by converting speaker information via information perturbation, the model can learn various types of speaker information, excluding those in the dataset. To evaluate the performance of the proposed method, we measured objective performance indicators, namely speaker encoder cosine similarity (SECS) and mean opinion score (MOS). To evaluate the synthesis performance for both the seen speaker and unseen speaker scenarios, Grad-TTS, SC-GlowTTS, and YourTTS were compared. The results demonstrated excellent speech synthesis performance for seen speakers and a performance similar to that of the zero-shot multi-speaker speech synthesis model.

## 1. Introduction

Speech synthesis is a technology that converts text into speech waveforms [1]. Speech synthesis technology is used in various application fields, such as artificial intelligence assistants, navigation systems, and audio books. Additionally, as speech synthesis technology is used in spoofing attacks on automatic speaker verification systems (ASVs), voice spoofing detection technology is being researched to prevent attacks through synthesized speech [2]. In order to detect spoofing attacks using synthetic speech, natural synthetic speech is needed as training data, and accordingly, research is being conducted to generate natural speech. With the development of deep learning, neural network-based speech synthesis technology is being researched in various fields, and the quality of synthesized speech has significantly improved. The majority of neural network-based speech synthesis models consist of two main steps. First, a feature generation model converts input text into acoustic features in the time–frequency domain. Second, a vocoder model synthesizes the acoustic features into raw waveforms [3].

An example of a neural network-based speech synthesis model is Tacotron, which uses an attention-based sequence-to-sequence structure [4,5]. Tacotron is an end-to-end model that directly generates speech without intermediate acoustic features. Tacotron uses attention technology to align the length between text and speech, thereby enabling more accurate speech synthesis. However, Tacotron has a slow synthesis speed because it uses an autoregressive method. In addition, because it relies on the duration of the attention module, there are problems with incorrect pronunciation or skipping phonemes. FastSpeech, a transformer-based model, utilizes a non-autoregressive approach to address these limitations, providing a fast inference speed [6]. Furthermore, estimating the alignment between the mel-spectrogram and text using the duration predictor makes it possible to compensate for incorrect pronunciation and achieve more natural speech synthesis. However, in learning the duration predictor, the alignment calculated from the autoregressive model is used as a label. Therefore, if the alignment calculated from the autoregressive model is not accurate, the duration predictor will estimate an incorrect alignment.

Glow-TTS, a speech synthesis model utilizing normalizing flows, introduced a monotonic alignment search (MAS) [7,8]. MAS aims to estimate the optimal monotonic alignment by leveraging that the alignment between the text and the mel-spectrogram is monotonic and that phonemes are not skipped. The MAS computes the log likelihood between the prior distribution of the text and the latent representation of the mel-spectrogram. Dynamic programming is then applied using the calculated log likelihood to estimate the monotonic alignment between the text and the mel-spectrogram. Speech produced in this manner can deliver robust speech without skipping or omitting phonemes.

Recently, models based on the denoising diffusion probabilistic model (DDPM) have demonstrated high performances in various domains [9]. DDPM is a generative model that generates data through a forward process, which gradually adds noise to the original data distribution to convert it into an easy-to-handle data distribution, and a backward process returns it to the original data. These DDPMs have demonstrated high performances in various domains. DaLLE2 and stable diffusion models exhibit high performance in the text-to-image field [10,11]. In the audio field, DiffWave models convert acoustic features into audio waveforms [12]. Grad-TTS is a successful DDPM-based speech synthesis model [13]. Furthermore, Grad-TTS uses stochastic differential equations to represent forward and backward processes and synthesizes speech using a score-based decoder [14]. A score-based decoder is a network that estimates a score at a particular time, where the score refers to the gradient of the log probability density of the data. Using the estimated scores, the data are generated using a numerical ordinary differential equation (ODE) solver. Grad-TTS can produce more natural speech than existing speech synthesis models, accept speaker ID as an input, and support multi-speaker speech synthesis. However, because it receives the speaker ID as the input, it is not possible to synthesize speech for unseen speakers, that is, speakers not registered in the dataset. This means that the model has less controllability over the speaker because it receives only limited information about the speaker as the input. Therefore, the zero-shot multi-speaker speech synthesis model, which can be used for unseen speaker speech synthesis, uses speaker embedding instead of speaker ID. Speaker embedding refers to speaker information extracted from speech using a speaker encoder for speaker recognition.

Speaker recognition is a technology that identifies speakers by extracting information from speech [15]. Neural network-based speaker recognition models perform speaker identification by extracting speaker embeddings from speech and calculating the similarity between vectors. Furthermore, models trained using large-scale speech datasets can learn diverse speaker information, thereby enabling speaker recognition by unseen speakers. A typical speaker recognition model is the ECAPA-TDNN [16]. The ECAPA-TDNN model improves the time delay neural network (TDNN) model and demonstrates excellent recognition performance in VoxSRC-2019 [17]. Recently, zero-shot multi-speaker speech synthesis using speaker recognition models has been researched, and studies have reported that the performance of zero-shot multi-speaker speech synthesis can be improved according to the performance of the speaker recognition model [18]. Therefore, models that support zero-shot learning typically use a speaker recognition model pre-trained on large-scale speech datasets as a speaker encoder to obtain speaker information.

Speech synthesis models that support zero-shot multi-speakers with speaker encoders can synthesize high-quality speech for various speakers with only a few seconds of speech referencing. Representative zero-shot multi-speaker speech synthesis models include SC-GlowTTS and YourTTS [19,20]. SC-GlowTTS, which performs speech synthesis based on the Glow-TTS model, extracts speaker information from the reference speech via a speaker encoder. The extracted speaker information is provided as a condition for generating the speaker’s speech. This allows for the synthesis of speech from a variety of speakers, as well as from unseen speakers. In addition, YourTTS, capable of end-to-end speech synthesis, is a zero-shot multi-speaker speech synthesis model based on VITS, which combines normalizing flow and VAE [21,22]. Furthermore, YourTTS is a model that enables zero-shot multi-speaker speech synthesis and multilingual training via language ID. However, zero-shot multi-speaker speech synthesis models have a limitation in that the performance of speech synthesis for unseen speakers is lower than that for seen speakers. SC-CNN proposed an effective speaker conditioning method to resolve the imbalance in speech synthesis performance between unseen speakers and seen speakers [23]. It conditions speaker embeddings in a way that considers the close correlation between adjacent phonemes and provides speaker-dependent local correlations. It provides superior zero-shot multi-speaker speech synthesis performance than conventional speaker conditioning methods. Another problem with the zero-shot multi-speaker speech synthesis models is that they have a limitation in that speech synthesis performance deteriorates in low-resource environments with minimal data. This problem can occur because of insufficient training data and the inability of the model to learn the language sufficiently. Therefore, research has recently been conducted to convert speaker information into speech data so that models can learn various types of speaker information.

Similarly, NANSY, which exhibits high performance in voice conversion, is a typical method that converts speaker information from speech and utilizes various types of information. NANSY introduced information perturbation, which distorts speaker characteristics and transforms speaker information to selectively control the desired features between the context and speaker information [24]. Information perturbation is a technique that perturbs speaker information by transforming the pitch, formant, and frequency shapes of the speech. Before NANSY, the voice conversion model AUTOVC utilized a method for adjusting the bottleneck dimension to control the speaker and context information [25]. This method requires extremely delicate adjustment of the bottleneck dimension, and if the bottleneck dimension is set incorrectly, voice conversion will not be performed accurately. NANSY proposed a perturbation-based voice conversion method that uses information perturbation to remove speaker-dependent information from speech, allowing the model to consider only contextual information.

In this study, we improved the existing Grad-TTS and proposed an effective zero-shot multi-speaker speech synthesis model. The existing Grad-TTS performs multi-speaker speech synthesis through speaker ID. However, this method has limitations in that controllability of speaker information is poor and speech synthesis is possible only for seen speakers. Therefore, in this paper, we aim to support zero-shot multi-speaker speech synthesis through speaker encoder and information perturbation and improve the controllability of speaker information and generalization performance of the model. The proposed method is based on the Grad-TTS structure and integrates information perturbation and a speaker encoder to guide the model in learning various types of speaker information. Specifically, speaker embeddings are extracted through a speaker encoder and used as conditions in the synthesis process. This allows the proposed method to support zero-shot multi-speaker speech synthesis. Moreover, we used the information perturbation introduced in NANSY to improve the speech synthesis quality of unseen speakers. In this study, we assume that information perturbation considers various types of speakers from the perspective of speech synthesis. Therefore, by using information perturbation, we can train the model to consider a wider range of speaker information, allowing it to learn more speakers than are included in the dataset. The main difference between the proposed method and Grad-TTS lies in the speaker information received. While Grad-TTS receives speaker ID, the proposed method receives speaker embeddings extracted from the reference speech. This allows the model to rely on reference speech during inference to reflect speaker information not only for seen speakers but also for unseen speakers. This can model speaker characteristics more effectively than speaker conditioning using existing speaker ID and improves the controllability of speaker information. Additionally, through information perturbation, the model is trained to consider more speaker information in addition to the speakers included in the dataset. Unlike the existing Grad-TTS training method, this allows the model to learn more speaker information, increasing speaker similarity to unseen speakers and improving the generalization performance of the model. To evaluate the speech synthesis performance of the speaker using the proposed method, comparisons were conducted with the baseline Grad-TTS method. Furthermore, to evaluate the synthesis performance of the unseen speaker, comparisons were made between SC-GlowTTS and YourTTS.

This paper is structured as follows. Section 2 describes the structure and learning method of the proposed method. Section 3 describes the dataset and experimental settings used to train the model and compares the performance of the models. Finally, Section 4 presents the conclusions of this paper.

## 2. Zero-Shot Multi-Speaker Text-to-Speech Technique

**Figure 1:**
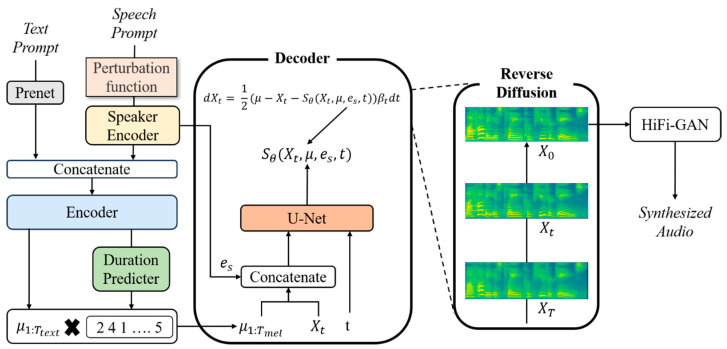
Overall structure of proposed zero-shot Grad-TTS.

Figure 1 shows the overall structure of the proposed model. The text prompt given as the input is converted to a symbol ID, and the speech prompt is used with the pitch, formant, and frequency shape randomly converted by the perturbation function. The speaker encoder shown in Figure 1 is a speaker recognition network pre-trained with VoxCeleb2, which extracts a speaker embedding $e_{s}$ that represents speaker information from the speech prompt [26]. PReNet is a network that extracts features from text expressed by symbol IDs, and consists of a convolution layer. The encoder is composed of a transformer. It takes the features extracted from the PReNet and the speaker embedding $e_{s}$ as the input to generate text embeddings $\mu_{1:T_{text}}$ that incorporate speaker information. To convert the text embedding generated in this manner into a mel-spectrogram via a decoder, it is necessary to consider the alignment between the text and mel-spectrogram sequences. A duration predictor is a network that considers the alignment of two features in different domains. It takes the text embedding $\mu_{1:T_{text}}$ as the input and estimates the duration for each text. The duration predictor used in this study was trained using the MAS algorithm proposed in Glow-TTS [8]. Using the estimated duration, it extends the length of the text embedding to create a text embedding $\mu_{1:T_{mel}}$ with the same sequence as the mel-spectrogram. $X_{t}$ in Figure 1 represents noisy data created by adding scheduled noise at a specific time t to the text embedding $\mu_{1:T_{mel}}$. U-Net receives noisy data $X_{t}$, time information t, speaker embedding $e_{s}$, and text embedding $\mu_{1:T_{mel}}$ as the inputs, and estimates the amount of noise added at time t [27]. The reason for estimating the data noise in this manner is that the distribution of the actual data is not known; therefore, it is difficult to obtain a score, which is the log probability density gradient for the data. Thus, the data score was approximated by adding noise to the data and determining the log probability density gradient of the noisy data. Using the estimated denoising score, the backward process is performed by solving the ODE, ultimately generating the target mel-spectrogram $X_{0}$. The estimated $X_{0}$ is generated as a speech waveform using the HiFi-GAN [28].

### 2.1. Speaker Encoder

To generate the speech of the target speaker, speaker information was extracted from the speech prompt and used as a condition for the model. The most common method for extracting speaker information from speech is to use a speaker recognition model trained on a large-scale dataset to extract information about the speaker from the target speech. Therefore, in this study, we used the ECAPA-TDNN model trained on approximately 2442 h of the VoxCeleb2 dataset as a speaker encoder to extract 192-dimensional speaker embeddings. The speech prompt given in the input was used by randomly cutting 5 s of speech from all the speeches and padding speech of 5 s or less. The speaker embeddings extracted in this manner were used as conditions for the encoder and decoder.

In zero-shot speech synthesis using a speaker encoder, speech for a seen speaker may occasionally be generated instead of speech similar to the reference speech. This is because, despite using a speaker encoder trained on a large-scale speaker dataset, decoding with text embedding and speaker embedding as the inputs ultimately leads to learning only for the seen speakers. The most effective method for addressing this drawback is to train the model using a large-scale speech dataset with a wide range of speaker information. This allows the model to learn a large amount of speaker information, which improves its generalization performance. However, speech synthesis models have complex structures consisting of various modules, making them large and expensive to train. In this study, we propose a method for improving the generalization performance of a model by adding an information perturbation technique. This technique helps prevent incorrect speech synthesis for unseen speakers by making the model more robust to speaker variations. The information perturbation technique proposed in NANSY perturbs the acoustic information of the speaker in the original speech by using formant shifting (fs), pitch shifting (ps), and random frequency shaping using a parametric equalizer (peq). Equations (1) and (2) describe the information perturbation techniques used in NANSY. Equation (1) applies fs, ps, and peq to speech, whereas Equation (2) applies fs and peq. In NANSY, Equation (1) was used to extract contextual information from the speech, and Equation (2) was used to extract the pitch information.

(1) $$f\left(x\right)=fs\left(ps\left(peq\left(x\right)\right)\right).$$

(2) $$g\left(x\right)=fs\left(peq\left(x\right)\right).$$

To enable the model to learn from various speaker information through information perturbation, 50% of each mini-batch was set to include speech with no applied equations, whereas the remaining 50% was set to apply either Equation (1) or Equation (2) randomly. This was completed to ensure that speech with speaker information was completely transformed, speech with pitch information was preserved, and the remaining acoustic features transformed were included, allowing for a variety of speech information.

### 2.2. Diffusion Model

The DDPM consists of a forward process that gradually adds noise to the data to converge itself into a Gaussian distribution and a backward process that removes noise from the Gaussian distribution to generate data [9]. In this study, we performed speech synthesis using a diffusion probabilistic model based on stochastic differential equations (SDE) following the approach introduced in Grad-TTS [13].

(3) $$dX_{t}=\frac{1}{2}\sum^{-1}\left(\mu -X_{t}\right)\beta_{t}dt+\sqrt{\beta_{t}}d{\vec{W}}_{t}$$

(4) $$dX_{t}=\left(\frac{1}{2}\sum^{-1}\left(\mu -X_{t}\right)-S_{\theta }\left(X_{t},\;\mu ,\;e_{s},t\right)\right)\beta_{t}dt+\sqrt{\beta_{t}}d{\overset{↼}{W}}_{t}.$$

Equations (3) and (4) are the SDE that represent the forward and backward processes used in the diffusion probabilistic models. μ is the encoder output generated by extracting context information from the text and speaker information from the prompt speech. This is an 80-dimensional vector of the same length as the target mel-spectrogram. $e_{s}$ refers to the 192-dimensional speaker embedding extracted using a speaker encoder. ${\vec{W}}_{t}$ and ${\overset{↼}{W}}_{t}$ are standard forward and backward Brownian motions, respectively. $\beta_{t}$ is a positive function that schedules noise at time t. In Equation (4), $S_{\theta }$ is a network that estimates the score of noisy data. It estimates the amount of noise in $X_{t}$ at time t.

(5) $$dX_{t}=\left(\frac{1}{2}\sum^{-1}\left(\mu -X_{t}\right)-S_{\theta }\left(X_{t},\;\mu ,\;e_{s},\;t\right)\right)\beta_{t}dt,$$

In this study, we perform a forward process, which adds noise to the data, using Equation (3). We also perform the backward process through an ODE that does not consider probabilistic properties such as Grad-TTS. Equation (5) represents the backward process, which is expressed as an ODE. Equation (5) is different from Equation (4) in that it does not contain $\sqrt{\beta_{t}}d{\overset{↼}{W}}_{t}$, which is a probabilistic diffusion process. This eliminates the probabilistic diffusion process and transforms the stochastic differential equation into an ODE. Consequently, the backward process is performed in a deterministic form. This method of performing the backward process has the advantage of a faster data sampling speed compared to Equation (4).

### 2.3. Loss Function

In this study, we trained three modules—the encoder, decoder, and duration predictor—for model learning. To learn this, we followed the training method used in the Grad-TTS. Equation (6) represents the objective function for encoder learning. $\mu_{Align\left(i\right)}$ is the aligned encoder output, where ‘align’ is an alignment function that obtains the alignment between the text and mel-spectrogram and is estimated by the duration predictor. The objective function minimizes the distance between the aligned encoder output and the target mel-spectrogram. This enables the decoder to start decoding from a state as close as possible to the target mel-spectrogram distribution at inference, which makes it easier for the decoder to generate data.

(6) $$L_{encoder}=-\sum_{i=1}^{F}\log\delta \left(y_{i};\;\mu_{Align\left(i\right)},\;I\right).$$

To train a duration predictor that estimates the alignment between the text and the mel-spectrogram, we need to obtain the alignment between the text and the mel-spectrogram as labels. Therefore, we obtained the alignment between the two domains using the MAS proposed in Glow-TTS and trained the duration predictor using the obtained alignments as labels. MAS is an algorithm that determines the optimal monotonic alignment using dynamic programming, assuming that the alignment between the text and mel-spectrogram is a monotonic function. However, optimizing the alignment function and encoder loss is difficult. Therefore, we learned this in two stages, as in the Grad-TTS. The learning approach is as follows. First, the encoder parameters are fixed, and the optimal alignment function is estimated. Subsequently, the Align function is fixed, and the encoder parameters are updated. Thus, the encoder and duration predictors were trained in stages.

Equation (7) uses the alignment function to calculate the number of mel frames for each phoneme. In Equation (7), the MAS estimates the Align function, and the duration predictor is trained using $d_{i}$ obtained from the equation as labels. Equation (8) is the objective function used to train the duration predictor. Furthermore, P denotes the duration predictor, a network that predicts the duration of phonemes, and sg denotes the stop gradient, which stops the gradient calculation. The reason for stopping the gradient calculation of μ is to prevent the encoder from being trained during the training of the duration predictor.

(7) $$d_{i}=\log\sum_{j=1}^{F}1_{Align^{*}\left(j\right)=i},\;\;\;\;\;\;\;\;\;i=1,\;\ldots ,\;l,$$

(8) $$L_{duration}=MSE\left(P\left(sg\left[\mu \right]\right),\;d\right).$$

Equation (9) is a diffusion loss function for training a score-based decoder. In Equation (9), $X_{0}$ denotes the target mel-spectrogram, and $X_{t}$ denotes the mel-spectrogram with noise diffused at a specific time t. $S_{\theta }$ is a network that estimates the amount of noise in $X_{t}$ at a randomly sampled time t in the interval [0, T]. This network learns to estimate the denoising scores.

(9) $$L_{diffusion}=E_{X_{0},\;\;t}\left[\lambda_{t}E_{\xi_{t}}\left[\|S_{\theta }\left(X_{t},\;\mu ,e_{s},t\right)+\frac{\xi_{t}}{\sqrt{\lambda_{t}}}{\|}_{2}^{2}\right]\right].$$

Finally, the encoder, decoder, and duration predictor modules are trained using the abovementioned loss functions. In addition, because the alignment function constantly changes during training, it is trained in multiple stages. The overall training process is as follows:

- Fix the encoder, decoder, and duration predictor modules, and find the alignment Align that minimizes $L_{encoder}$ using the MAS algorithm.
- Fix the alignment Align and minimize the loss for $L_{encoder}+L_{duration}+L_{diffusion}$.
- Repeat the above two steps.

## 3. Experiments

### 3.1. Experimental Setup

We trained the model using the LibriTTS dataset, a multi-speaker speech synthesis dataset with approximately 555 h of English speech [29]. The dataset comprises 2311 speakers, including 1198 female and 1113 male speakers. Each speaker has a corresponding text transcription file for their speech. In addition, we used the VCTK Corpus dataset, which consists of approximately 44 h of speech from 109 speakers, as the test dataset to evaluate the performance of unseen speakers [30].

Although the LibriTTS dataset is provided at 24 kHz, the individual modules used in this study were pre-trained models that were trained at different sampling rates. Therefore, it was necessary to convert the sampling rates accordingly. Since HiFi-GAN was trained at a sampling rate of 22.05 kHz and the ECAPA-TDNN was trained at a sampling rate of 16 kHz, they were downsampled to 22.05 kHz and 16 kHz, respectively. The downsampled speech was converted into 80-dimensional mel-spectrograms, which were used as the input to the speaker encoder and labels for the model. In addition, the window size and hop length used for the conversion were set to 1024 and 256, respectively. We used the Adam optimizer for training at a learning rate of 2 × 10^−4^. As a result of this training, the proposed model ended training at 130 epochs, and the final loss was about 0.506 for duration loss, about 1.365 for encoder loss, and about 0.111 for diffusion loss.

We evaluated the performance of the proposed model using two objective evaluation metrics: speaker encoder cosine similarity (SECS) and mean opinion score (MOS). SECS is an evaluation metric measuring speaker similarity between generated and original speech. It measures the similarity between speaker embeddings extracted from the generated and original speech by inputting them into a speaker encoder. SECS has a range of −1 to 1, with higher values indicating a higher similarity. The mean opinion score (MOS), a perceptual evaluation metric, is a subjective method for measuring the quality of the speech generated. Furthermore, it requests multiple participants to rate the quality of synthesized speech, such as its naturalness and similarity to the reference speech. These MOSs are widely used as evaluation metrics in speech synthesis models. However, a MOS evaluation requires many participants, which is time consuming and costly. Hence, these limitations have led to an increasing trend in using neural MOS predictors to evaluate the MOS in recent years. Therefore, this study uses a pre-trained MOS predictor, SSL-MOS, to evaluate the naturalness of speech. In addition, the MOS predictor selected was employed as a baseline in the 2022 VoiceMOS Challenge [31,32].

To evaluate the performance of the proposed model on the seen speaker speech synthesis, we compared it with the baseline Grad-TTS model. We used the same parameters for the inference tasks to ensure a fair comparison. In addition, we compared the zero-shot speech synthesis models SC-GlowTTS and YourTTS based on flow to measure the synthesis performance for unseen speakers. The audio samples used for the performance evaluation are available on the demo website (https://github.com/cjchun3616/zero_shot_gradtts/, accessed on 23 October 2023).

### 3.2. Experimental Result

**Figure 2:**
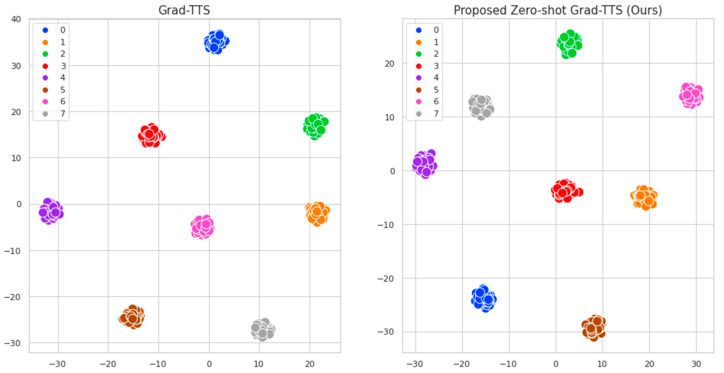
t-SNE analysis results of Grad-TTS and proposed zero-shot Grad-TTS (this study).

Table 1 shows the SECS scores of the synthesized speech for seen speakers from the proposed model and Grad-TTS. To measure the performance, we randomly selected 20 speakers from the LibriTTS dataset (10 males and 10 females) and generated five samples for each type of speakers. We extracted embedding vectors from the speaker encoder for the generated samples and the reference speech and then measured the cosine similarity between the generated and reference speech. As shown in Table 1, Grad-TTS achieved a higher cosine similarity (0.8907) than the proposed model. Figure 2 shows the t-SNE analysis results for speaker embeddings extracted from the synthesized speech generated by Grad-TTS and the proposed model. Figure 2 shows that the speaker embeddings of the same speaker are clustered together for both Grad-TTS and the proposed model. The results of the t-SNE analysis suggest that the proposed model produces audio that sufficiently expresses speaker information, similar to Grad-TTS, despite having a lower cosine similarity than Grad-TTS. Table 2 lists the MOS results obtained using the SSL-MOS model. The evaluation results demonstrate that the proposed model produces more natural speech than Grad-TTS, with an MOS score of 4.39 ± 0.16. Therefore, the proposed model can still generate natural speech for the seen speaker and produce speech with speaker similarity levels similar to Grad-TTS despite supporting zero-shot multi-speaker speech synthesis.

To evaluate the speech synthesis performance of unseen speakers in this study, we compared the synthesized speech quality of the proposed model with two other flow-based zero-shot multi-speaker speech synthesis models, SC-GlowTTS and YourTTS. For speech synthesis, we used the 5th utterance in the VCTK dataset as the reference speech, and 55 utterances were randomly selected for the text to be synthesized. To measure the performance, we selected 11 speakers that were not used for training by any of the three models and performed the evaluation. The results are summarized in Table 3. It is evident that the proposed model achieved the highest speaker similarity of 0.4020 among the three models for SECS, which measures the similarity between speakers. Table 4 shows the results of measuring the SSL-MOS, which evaluates the naturalness of speech. According to the SSL-MOS measurement results, YourTTS recorded the highest score of 4.52 ± 0.07, followed by the proposed model with a score of 4.33 ± 0.09. The results show that the proposed model can generate audio with higher speaker similarity than zero-shot multi-speaker models in a zero-shot environment; although it achieves a lower MOS than YourTTS, it can generate natural audio compared to SC-GlowTTS.

### 3.3. Ablation Study

In this paper, an ablation study was performed to confirm the improvement of model generalization performance through information perturbation. For this purpose, we performed a SECS evaluation between a model that learned by applying perturbation and a model that did not apply perturbation. The samples generated for the evaluation used the VCTK dataset, which represented unseen speakers. The text prompt used to generate the samples was the same as the prompt used in Table 3, and the speech prompt used the 6th sample from each type of speakers. Table 5 shows the cosine similarity scores with and without information perturbation. As a result of the evaluation, the model applying perturbation showed the highest cosine similarity of 0.3989 for unseen speakers. These evaluation results indicate that information perturbation is effective in improving speaker similarity for unseen speakers. In other words, it shows that information perturbation is effective in improving the generalization performance of the model by considering various speaker information, as assumed in this paper.

## 4. Conclusions

This study proposed a method to improve Grad-TTS for effective zero-shot multi-speaker speech synthesis using a speaker encoder and information perturbation. The proposed method extracts speaker information from reference speech using a speaker encoder and uses it as the input to the model. This allowed the model to receive information from various speakers, making it possible to synthesize speech for unseen speakers. In addition, the proposed method applies information perturbation to the speaker information to make it more diverse, allowing the model to learn a wider range of speaker information. The evaluation results show that the proposed model has a slightly lower speaker similarity than the baseline Grad-TTS. However, the t-SNE analysis results showed that the proposed model generated speech that expressed speaker information like that of Grad-TTS. Furthermore, the proposed model also achieved a higher MOS score than Grad-TTS in the MOS evaluation. This indicates that the proposed model, while supporting zero-shot multi-speaker speech synthesis, maintained its speech synthesis performance for the seen speakers. In addition, the proposed model achieved a slightly higher speaker similarity than YourTTS in the speech synthesis performance evaluation for unseen speakers. In the MOS evaluation, the proposed model achieved the second-highest MOS score. This shows that the proposed model can generate speech with relatively high speaker similarity in a zero-shot environment compared to the existing zero-shot multi-speaker speech synthesis models. This also shows that the proposed model can generate more natural speech than SC-GlowTTS. As a result of this experiment, we were able to confirm that the proposed model supports zero-shot multi-speaker speech synthesis and shows high speech synthesis quality for unseen speakers.

## Figures and Tables

**Table 1:** SECS results of synthesized speech for seen speakers.

Model	SECS
Grad-TTS	0.8907
Ours	0.8849

**Table 2:** SSL-MOS results of synthesized speech for seen speakers.

Model	SSL-MOS
Grad-TTS	4.14 ± 0.15
Ours	4.39 ± 0.16

**Table 3:** SECS results of synthesized speech for unseen speakers.

Model	SECS
SC-GlowTTS	0.3400
YourTTS	0.4013
Ours	0.4020

**Table 4:** SSL-MOS results of synthesized speech for unseen speakers.

Model	SSL-MOS
SC-GlowTTS	2.66 ± 0.17
YourTTS	4.52 ± 0.07
Ours	4.33 ± 0.09

**Table 5:** SECS results of synthesized speech with and without information perturbation applied.

Model	SECS
Ours without perturbation	0.3801 ± 0.0558
Ours with perturbation	0.3989 ± 0.0479

## Data Availability

The data generated from the simulation is accessible on https://github.com/cjchun3616/zero_shot_gradtts/ (accessed on 23 October 2023).
